# Updates in Reproduction Coming from the Endocannabinoid System

**DOI:** 10.1155/2014/412354

**Published:** 2014-01-16

**Authors:** Rosaria Meccariello, Natalia Battista, Heather B. Bradshaw, Haibin Wang

**Affiliations:** ^1^Dipartimento di Scienze Motorie e del Benessere, Università di Napoli Parthenope, via Medina 40, 80133 Napoli, Italy; ^2^Faculty of Bioscience and Technology for Food, Agriculture and Environment, University of Teramo, 64100 Teramo, Italy; ^3^European Center for Brain Research (CERC), Santa Lucia Foundation, 00143 Rome, Italy; ^4^Department of Psychological and Brain Sciences, The Kinsey Institute for Research in Sex, Gender, and Reproduction, Indiana University, Bloomington, IN 47405, USA; ^5^State Key Laboratory of Reproductive Biology, Institute of Zoology, Chinese Academy of Sciences, Beijing 100101, China

## Abstract

The endocannabinoid system (ECS) is an evolutionarily conserved master system deeply involved in the central and local control of reproductive functions in both sexes. The tone of these lipid mediators—deeply modulated by the activity of biosynthetic and hydrolyzing machineries—regulates reproductive functions from gonadotropin discharge and steroid biosynthesis to the formation of high quality gametes and successful pregnancy. This review provides an overview on ECS and reproduction and focuses on the insights in the regulation of endocannabinoid production by steroids, in the regulation of male reproductive activity, and in placentation and parturition. Taken all together, evidences emerge that the activity of the ECS is crucial for procreation and may represent a target for the therapeutic exploitation of infertility.

## 1. Introduction

**Figure 1:**
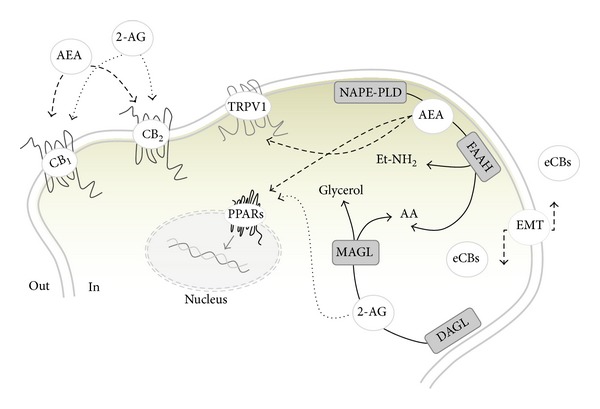
Schematic representation of the ECS. *N*-arachidonoyl-ethanolamine (AEA) is mainly produced by the activity of an *N*-acylphosphatidylethanolamine-specific phospholipase D (NAPE-PLD), whereas its degradation is due to a fatty acid amide hydrolase (FAAH), which releases ethanolamine (Et-NH_2_) and arachidonic acid (AA). 2-Arachidonoylglycerol (2-AG) is also released from membrane lipids through the activity of diacylglycerol lipase (DAGL) and can be hydrolyzed by a cytosolic monoacylglycerol lipase (MAGL) that releases glycerol and AA. The cellular uptake from the extracellular to the intracellular space is ascribed to a purported “endocannabinoid membrane transporter (EMT)” that is likely to take up both AEA and 2-AG. Both eCBs trigger several signal transduction pathways by acting at their targets, CB_1_, CB_2_, GPR55, and nuclear peroxisome proliferator-activated receptors (PPARs). AEA, but not 2-AG, binds intracellularly also Transient Receptor Potential Cation Channel type 1 (TRPV1).

Reproductive functions are under a fine regulation exerted at multiple levels along the hypothalamic-pituitary-gonadal axis. The formation of high quality gametes, followed by a successful pregnancy event, is the result of deep cell to cell communications. In this respect, the list of potential modulators of reproductive activity is still growing. In the last two decades the upcoming role of lipid mediators that share some of the effects with delta-9-tetrahydrocannabinol (Δ^9^-THC), the active principle of marijuana plant, *Cannabis sativa, *emerged. These bioregulators, collectively named endocannabinoids (eCBs), are amides, esters, and ethers of long-chain polyunsaturated fatty acid and have been detected in most reproductive tissues and fluids [1–3].Besides ligands, a wide range of receptors, biosynthetic and hydrolyzing enzymes, and putative membrane transporters (EMT) all together form the endocannabinoid system (ECS) (Figure 1), a master system that is deeply involved in the central and local control of male and female reproduction. Since their discovery, research made giant strides in the comprehension of physiological, cellular, and molecular events in reproduction driven by eCBs. Many inputs in the field came from studies conducted in invertebrates and nonmammalian vertebrates, indicating that ECS is an evolutionarily conserved master system deeply involved in the control of reproductive functions. Thus, the aim of this review is just to provide new insights into the complex field of eCBs and reproduction.

## 2. ECS and Reproduction: An Overview

Smoking marijuana has always represented a warning for the long lasting effects not only on physical and mental performances but also on the reproductive events. The effects of Δ^9^-THC on pregnancy was highlighted for the first time at the beginning of 1970 [4] and, since then, numerous papers have been focused on the potential aversive effects caused by the use of recreational drugs during gestation and in offspring born from cannabis users.

In 1992, *N*-arachidonoylethanolamine (also known as anandamide, AEA), a cannabinoid-like compound, was identified to compete with exocannabinoid ligands for type 1 and type 2 cannabinoid receptors (CB_1_ and CB_2_, resp.) [5, 6] and, few years later, the group of Dr. Schuel and Dr. Das reported the ability of AEA to affect negatively both male and female fertility [7, 8]. In the next years, the endocannabinoid signaling was demonstrated to play a key role in the preimplantation embryo development [9–12] and it was supposed that AEA content could be critical for a timely embryo implantation [13]. Nowadays, it is clear that, in order to guarantee a receptive uterine environment, AEA levels must be kept low [14], and this is accomplished through a tight regulation mediated by an  *N*-acylphosphatidylethanolamine-specific phospholipase D (NAPE-PLD), the enzyme responsible for its synthesis, and fatty acid amide hydrolase (FAAH), in charge of its degradation to arachidonic acid and ethanolamine [15–18]. Further confirmations on the harmful effects caused by high AEA levels for a normal pregnancy outcome were obtained from measurements of AEA levels in plasma samples from women with normal menstrual cycle and laboring patients [19] and NAPE-PLD and FAAH analysis performed on human placenta [20].

In the same timeframe, several experimental studies highlighted the ability of AEA in regulating sperm functions required for fertilization [7, 21], by reducing sperm motility, inhibiting capacitation-induced acrosome reaction and mitochondrial activity [21, 22]. Cannabinoid and vanilloid (in particular transient receptor potential cation channel type 1, TRPV1) receptors mediate the physiological action of AEA with double effects. On the one hand, AEA binding to CB_1_ affects the capacitation process in mammals [23–25]; on the other hand activation of the intracellular site of TRPV1 inhibits spontaneous acrosome reaction in porcine [23] and human sperm cells [26]. Lately, TRPV1-mediated activities of AEA were also reported in capacitated mouse spermatozoa (SPZ), where elevated intracellular levels of AEA are due to a reduced FAAH activity [27]. In the last decade, many studies have been focused on the involvement of the CB_1_/CB_2_-signaling in follicle maturation, oviductal-uterine embryo migration, implantation and (neuro)development, placentation, and parturition onset, showing that any aberration of endocannabinoid signaling can severely affect these processes (for a review see [28]). Specific and selective antagonists of CB_1_/CB_2_ and/or CB_1_/CB_2_ knockout mice (*CB*_1_^−/−^ and *CB*_2_^−/−^, resp.) have always been useful tools that allowed researchers to better understand which target is critical to achieving correctly all reproductive events from sperm-oocyte fusion to the birth of offspring. In this context, we should recall that short and long term exposure to HU210, a selective agonist for CB_1_ and CB_2_, showed how the deregulation of the ECS markedly reduces total sperm count, depletes spermatogenic efficiency, and impairs sperm motility [29]. A recent paper by Fonseca and workers proposed a functional role of GPR55 receptor in the uterine remodeling and in immune processes activated during fetoplacental development [30]. The differential spatiotemporal expression pattern of GPR55 found in decidual and natural killer (NK) cells might implicate possible interactions of this target with other endocannabinoid-like compounds (i.e., *N*-palmitoylethanolamide), since the main eCBs lack affinity for this receptor [31].

Besides AEA, 2-arachidonoylglycerol (2-AG) is the other main representative of this family of bioactive lipids and its role in fertility seemed unknown up to few years ago, when its impact on mouse spermatogenesis [32], fetoplacental development [33], epididymal start-up [34], and mouse sperm capacitation [27] has been remarked. In fact, it has been reported that transcriptional and translational levels of 2-AG synthesizing (diacylglycerol lipase, DAGL) and hydrolyzing enzymes (monoacylglycerol lipase, MAGL) are finely tuned in various processes of male and female reproduction. This metabolic equilibrium is required in order to guarantee an appropriate 2-AG tone in reproductive stages; in this context, low 2-AG levels were detected in seminal plasma of infertile men, suggesting a reduced sperm fertilizing capacity through a mechanism yet to be explored [35].

**Figure 2:**
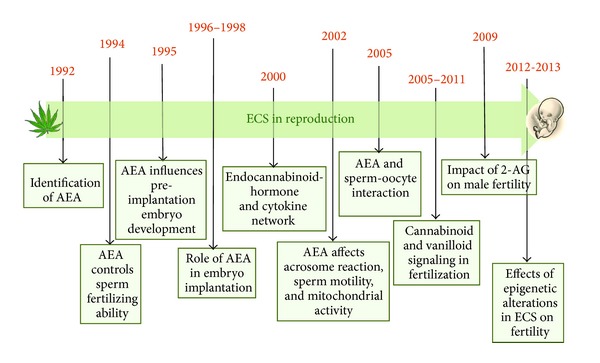
Major breakthroughs in male and fertility reproduction.

To date, we have good knowledge about the existence of a definite network, including eCBs, hormones, prostaglandins, and cytokines that warrant a successful pregnancy in animals and humans. In particular, the involvement of the eCBs in lymphocyte-mediated control of the hormone-cytokine crosstalk at the fetal-maternal interface was reported for the first time by the group of Dr. Maccarrone, showing that FAAH activity and protein were lower in women who miscarried and who underwent IVF treatment [36–38], whereas cannabinoid receptor binding and AEA-carrier were not altered during gestation [36, 39, 40]. Moreover, it seems that steroid hormones primarily regulate AEA levels, with estradiol (E_2_) increasing the levels and progesterone suppressing them, and that an *equilibrium* between profertility Th2 cytokines and antifertility Th1 cytokines is requested to establish blastocyst implantation, trophoblast growth, and pregnancy maintenance. On the male side, follicle stimulating hormone (FSH) regulates the expression of FAAH in Sertoli cells through an estrogen-mediated pathway [41], and, in turn, E_2_ levels induce, directly or indirectly, epigenetic modifications at the *FAAH* promoter site [42] and influence, via CB_1_ [43], chromatin remodeling of spermatids with a clear impact on spermatogenesis [44, 45]. A schematic chronological overview of local activity of eCBs in male and female reproduction is depicted in Figure 2.

## 3. Evolutionary Aspects of ECS

The study of physiological mechanisms by comparative approach is a fundamental tool to build general models. Key events in reproduction such as the activity of estrogen—classical female hormone—in spermatogonial proliferation [46] or nongenomic action of steroids themselves have been firstly discovered in nonmammalian species and then confirmed in mammals [47, 48].

In this respect, enzymes involved in endocannabinoid biosynthesis and/or degradation occur throughout the animal kingdom including deuterostomian (i.e., sea urchin), protostomian (i.e., crustaceans and nematodes), and basal (i.e., cnidarians and placozoans) invertebrates [49]. Conversely, molecular cloning of *CB*_*1*_ */CB*_*2*_ receptor orthologs has produced positive results only in urochordates (the sea squirt, *Ciona intestinalis*), in cephalochordata (the amphioxus, *Branchiostoma floridae)*, in nonmammalian vertebrates (fish, amphibians, reptiles, and birds), and in mammals, with duplication of *CB*_*1*_ or *CB*_*2*_ genes found in fish [49–51]. Thus, given that CB_1_/CB_2_ are unique to chordates, the molecular nature of endocannabinoid signaling in noncordate invertebrate is currently under investigation and may be related to primitive neuronal functions; conversely, the appearance of multiple receptors and receptor splicing forms coming from invertebrates to humans may indicate the subsequent occurrence of functional partitioning. However, the recent identification of candidate *TRPV1* orthologs in the genome of the sea urchin *Strongylocentrotus purpuratus* [52] and of the annelid, the leech *Hirudo medicinalis* [53], confirms the existence of an ancient non-CB_1_/CB_2_-mediated endocannabinoid signaling.

The functional conservation of ECS is not limited to the central nervous system (CNS) but also extends to the modulation of gonadal functions. The first direct evidence of endocannabinoid activity on male reproduction came from studies conducted in sea urchin to assess the mechanisms of acrosome reaction and polyspermy ([54] for review). In this respect, the endocannabinoid-signaling similarity in neurotransmitter release and acrosomic granule exocytosis let Meizel in 2004 speculate that the sperm may be a “neuron with a tail” adapted to fertilize egg cell [55]. However, in the last 10 years, evidences of endocannabinoid activity have been provided in testis and/or sperm of both invertebrates and vertebrates, including sea urchin, fish, frogs, mice, rats, boars, bulls, and humans [7, 21, 23, 26, 56–63]. AEA inhibitory effects on sperm motility and acrosome reaction have been conserved from sea urchin to mammals and elsewhere properly reviewed [54, 56]. A retrograde AEA signaling is involved in sperm-egg interaction in sea urchin [54], whereas CB_1_ and/or CB_2_ are differentially expressed in fish [64, 65] and frog ovary [60]. Interestingly, CB_1_ signaling is likely involved in the process of testicular regression in the gilthead seabream, *Sparus aurata*, a hermaphrodite species in which the gonadal tissues first develop as testes and then as functional ovary [66]. As described in paragraph 6, in mammals—human included—most female reproductive events, from ovogenesis and fertilization to successful pregnancy and parturition, require a functional endocannabinoid signaling, once again confirming the conservation of functions related to reproduction.

## 4. eCBs, Hypothalamic GnRH, and Steroids

Three main lines of evidence suggest that the eCBs and gonadal hormone signaling systems interact. (1) eCBs and their receptors are present throughout the hypothalamic-pituitary-gonadal (HPG) axis, (2) changes to the ECS cause changes in the HPG and changes in the HPG axis can affect the expression of eCBs, and (3) the ECS mediates behaviors, which are also mediated by gonadal hormones, such as motivation or reproduction (reviewed in [67]).

The CB_1_ partial agonist, Δ^9^-THC, has been implicated in negative reproductive outcomes, including the inhibition of ovulation in women [68] and lower serum luteinizing hormone (LH) and testosterone (T) in men [69]. The inhibitory effects of eCBs on gonadal hormone production suggest that eCBs help regulate this circuitry. Leydig cells in the testes contain CB_1_. *CB*_1_^−/−^ mice show reduced serum T levels and abnormal Leydig cell development. These results suggest that endocannabinoid signaling is essential for the organization of the reproductive system [70]. Upcoming observations in the hypothalamic control of reproductive functions and gonadal sex steroid production are described below.

### 4.1. Insights into the Hypothalamic Control of GnRH Activity

In the CNS eCBs are well known retrograde signals that modulate neuronal communications inhibiting presynaptic release of neurotransmitters including *γ*-aminobutyric acid (GABA) and glutamate. Postsynaptic synthesis of 2-AG or AEA is a phylogenetically widespread phenomenon described from mammals to annelids [49] which modulates neural activity through cannabinoid or vanilloid receptors. Brain maps of CB_1_, CB_2_, and TRPV1 have been provided in fish, amphibians, and mammals [57, 58, 71–73], with CB_1_/TRPV1 colocalization in specific hypothalamic nuclei in mammalian brain [72]. A master system in the central control of reproductive activity is the gonadotropin releasing hormone (GnRH), a hypothalamic decapeptide responsible for gonadotropin discharge and steroid biosynthesis [47, 48, 74]. Inhibitory effects of phytocannabinoids, cannabinoids and eCBs upon the endocrine control of reproduction have been largely described in the literature [51, 75]. Immortalized neuronal cell lines (GT1) possess a complete ECS and are themselves targets of endocannabinoid signaling, since the *in vitro* activation of cannabinoid receptors suppresses the pulsatile release of GnRH [76]. Furthermore, in the mediobasal hypothalamus of male rats, AEA intracerebroventricular injection suppresses GnRH release [77]. The importance of CB_1_ in negative modulation of reproductive axis has been demonstrated by altered GnRH signalling in *CB*_1_^−/−^ mice [44]. However, 2-AG is able to suppress LH secretion in wild-type but not in *CB*_1_^−/−^ mice [78], whereas AEA decreases LH levels also in *CB*_1_^−/−^ [78]. Thus, receptors other than CB_1_—that is, TRPV1—might be involved in such a modulation.

**Figure 3:**
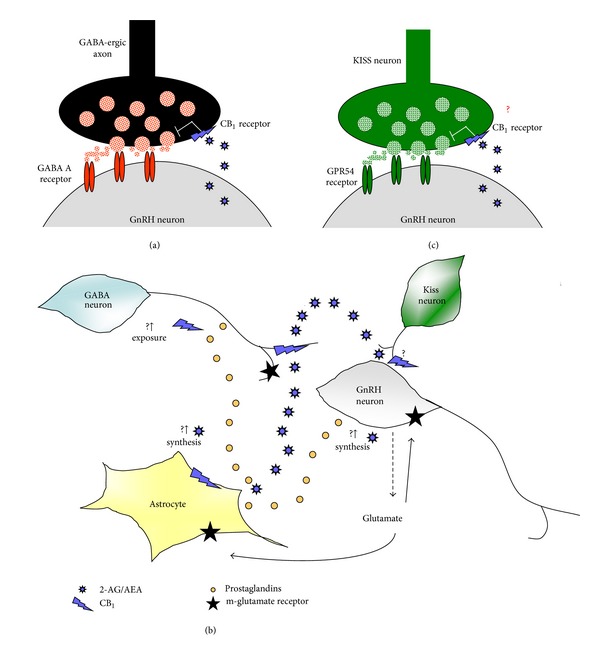
Possible mechanisms for the modulation of GnRH secreting neurons by eCBs. (a) GABAergic modulation of GnRH secreting neurons. GnRH secreting neurons release eCBs that, as retrograde signals, directly act on CB_1_ located on GABAergic presynaptic neurons and inhibit the release of GABA; as a consequence, GnRH secreting neurons do not receive GABAergic input and do not release the GnRH. (b) Possible involvement of glial cells in eCBs/GABA/GnRH circuitry. Glutamate release by GnRH neurons may stimulate astrocytes to produce prostaglandins which in turn induce the synthesis of eCBs and/or the exposure of presynaptic CB_1_, thus modulating GABA release. (c) Hypothesis: are there neuronal systems other than GABAergic able to modulate endocannabinoid/GnRH crosstalk? Kisspeptins stimulate gonadotropin discharge in several species modulating the activity of GnRH secreting neurons via the activation of GPR54 receptor located on GnRH neurons. Might AEA also act as retrograde signal upon kisspeptin neurons in order to suppress GnRH secretion?

Despite these observations, only recently has the mechanism involving direct/indirect endocannabinoid activity on the hypothalamic GnRH secreting neurons been provided. From fish to mammals GABA is a modulator of GnRH secreting neurons in the adult ([79, 80] and references inside). Metabolic, sex steroid, and circadian cues are usually conveyed to the GnRH system; involvement of metabotropic glutamate receptor located on astrocytes [81], eCBs [80] or GnRH itself [82] has been described in these routes. A 2-AG dependent inhibitory activity on the release of GnRH has been recently proposed in male mice [80]. At the molecular level, GnRH secreting neurons release 2-AG that directly acts as a retrograde signal on CB_1_ receptor located on GABAergic presynaptic neurons and inhibits the release of GABA (Figure 3(a)). As a consequence, GABA receptors located on GnRH secreting neurons are not activated and GnRH is not released [80]. Since astrocytes express CB_1_ [83] and eCBs can alter astrocyte transmitter uptake [84], a simplified alternative mechanism involving endocannabinoid-dependent modulation of glial cell functions (i.e., prostaglandin production) has been postulated [85]. In such a model, glutamate release by GnRH neurons may stimulate astrocyte to produce prostaglandins; these, in turn, may induce the synthesis of eCBs and/or the exposure of presynaptic CB_1_, thus modulating GABA release (Figure 3(b)).

Functional crosstalk among eCBs and GnRH neuronal systems has been described also in fish and amphibians [51, 57, 86–88], indicating that this is an evolutionarily conserved master system. In nonmammalian vertebrates, GnRH saga is more intricate since, as in humans, at least two distinct GnRH molecular forms (i.e., GnRH-I and GnRH-II) and one GnRH receptor (GnRH-R) have been described [47]. New insights in the central control of male reproduction emerged from nonmammalian vertebrates. CB_1_ has been localized in fish forebrain—the encephalic area mainly involved in the control of GnRH secretion and gonadotropin discharge— but in teleosts colocalization was observed in GnRH-III secreting neurons [57]. In the diencephalon of the anuran amphibian the frog *Rana esculenta*, CB_1_ dependent modulation of GnRH system expression rate (both ligands and receptors) has been reported [87, 88]. In particular, in the basal hypothalamus, *via* CB_1_, AEA significantly decreases *GnRH-I *and *GnRH-II *expression and upregulates *GnRH-RI* and *GnRH-RII* mRNA without any effect upon *GnRH-RIII * [87]. Twenty percent of hypothalamic GnRH-I secreting neurons possess CB_1_, and buserelin, a long acting GnRH analog, increases *CB*_*1*_ expression and inhibits those of *GnRH-I *[88]. The opposite profiles of CB_1_ and GnRH-I proteins [86] seem to confirm such AEA-dependent self-modulation route in which GnRH secreting neurons might produce eCBs to suppress the production of GnRH.

Conversely, as in the mouse, most frog GnRH-I secreting neurons are surrounded by CB1 immunopositive fibers, [76, 88] confirming the conservation of endocannabinoid-dependent retrograde signalling. GABAergic transmission, however, is not the only neuronal system that might be involved in the modulation of endocannabinoid/GnRH crosstalk. In this respect, one of the possible candidates is the kisspeptin signaling system. Kisspeptins, RFamide peptides encoded by the *kiss1* gene, stimulate LH and, to a lesser extent, FSH secretion in several species modulating the activity of GnRH secreting neurons *via* the activation of GPR54 receptor located on GnRH neurons [89]. Preliminary observations in male frogs indicate that *in vivo* administration of AEA suppresses the expression of diencephalic GPR54, turning off the GnRH system and steroidogenesis (Meccariello R., personal communication). Thus, it is not excluded that AEA might also act as retrograde signal upon kisspeptin neurons (Figure 3(c)). Interestingly, in both male and female, kisspeptin neuronal activities are strongly involved in steroid-dependent feedback mechanisms [90, 91].

As described in the next paragraph, AEA-dependent suppression of GnRH release is reversed by E2 administration in female rats [77] whereas, steroids represent the major factor in negative feedback mechanisms in males. Thus, in addition to E2-dependent modulation of endocannabinoid tone via FAAH modulation [42], the investigations concerning the possible crosstalk between kiss/GnRH/cannabinergic neurons [92] might open new insights in the molecular mechanisms of gonadal steroid feedback.

### 4.2. Interplay between Sex Steroids and eCBs

In the HPG axis, CB_1_ regulates sex hormone production. Intracerebroventricular injection of AEA reduced GnRH release in male and ovariectomized (OVX) female rats. However, in the same experiment, estrogen treated females experienced increased plasma LH after AEA injection. Therefore, estrogen possibly reverses the inhibitory effects of AEA [77]. Steroid hormones regulate CB_1_ expression in the pituitary [93]. In the rat pituitary there are sex differences; however the same may not be the case for humans. Therefore, in humans, nonsteroid signaling molecules may influence *CB*_*1*_ expression in the pituitary [94]. The possible direct activity of eCBs upon pituitary gland—evaluated in terms of ECS characterization as well as of eCBs dependent secretion of anterior pituitary hormones—has been suggested in vertebrates, but this issue is still controversial since species specific activities have been observed (for a recent review see [51]). In addition to the pituitary, cannabinoids and gonadal steroid function are linked in the hypothalamus. GnRH neurons in the medial preoptic area can synthesize endogenous cannabinoids, which exhibit negative feedback on GnRH release. However, it has also been noted that relatively few GnRH neurons contain *CB*_*1*_ mRNA, so eCBs must be exerting influence over neighboring cells [76]. Providing further evidence for a link between eCBs and sex hormones, endocannabinoid levels in the rat hypothalamus have been shown to fluctuate over the hormonal cycle. AEA levels reached a maximum during diestrous in the hypothalamus. Also, males showed significantly lower levels of 2-AG than females [95].

In a relationship critical to female reproductive success, an estrogen response element exists in the *FAAH* gene sequence. When estrogen binds to this response element, *FAAH *gene transcription is downregulated and AEA levels should remain elevated [96]. High doses of estrogen can have an anxiolytic effect. Hill et al. proposed that the anxiolytic effect is mediated by alterations in FAAH [97]. It may be that eCBs regulate the onset of puberty. eCBs may contribute to the peripubertal inhibition of GnRH neurons. Lopez hypothesizes that estrogen release from the ovaries at the time of puberty helps remove the endocannabinoid “brake” on reproductive functioning [69]. The overall relationship between estrogen and eCBs can be described as “bidirectional.” In one direction, endocannabinoid activity downregulates HPG axis activity, leading to reduced estrogen levels. In contrast, decreasing FAAH activity and modulating CB_1_ expression, estrogen up- regulates AEA production [67].

In addition to estrogen interactions, endocannabinoid activity attenuates progesterone release from the corpus luteum. Administration of AEA to pregnant rats caused a decrease in serum progesterone, as well as serum LH. Therefore, it appears that eCBs regulate the release of progesterone in two ways: (1) by directly binding onto receptor sites in the corpus luteum and (2) by directly controlling LH release in the CNS [98]. Like estrogen, progesterone can interact with a promoter region in the *FAAH* gene in that progesterone has been shown to increase *FAAH* expression in T-cells and human lymphoma U937 cells. In contrast, progesterone had no effect on *FAAH* expression in human neuroblastoma CPH100 cells [99]. Blocking progesterone receptors with antisense oligonucleotides eliminated the facilitating effect of Δ^9^-THC on female rodent mating behavior. In addition, blocking CB_1_, using SR141716, and blocking dopamine (DA) D1 receptors, using antisense nucleotides, also eliminated the effects of Δ^9^-THC on mating behavior [100]. Therefore, an interaction between progesterone, DA signaling, and cannabinoid signaling is necessary for female reproductive behavior. It is not known whether this could apply to human females.

As an example of how the ECS is involved in sexual motivation that is driven by sex steroids, studies with phytocannabinoids have been shown to affect sexual motivation. For example, exogenous CB_1_ agonist treatment in male rodents attenuates both appetitive and consummatory aspects of sexual behavior. However, studies in humans have been less conclusive. Men who use marijuana show great variation in sexual response [67]. For females, the effects of cannabinoids on sexual motivation and performance are much less clear. It appears that acute blocking Δ^9^-THC administration in female rats increased sexual receptivity at lower doses but decreased sexual receptivity at higher doses [101]. These findings are similar to Δ^9^-THC's effects on anxiety. Estrogen and DA have a complex relationship. Estrogen enhances dopaminergic activity in the nucleus accumbens via enhanced DA release and downregulates autoreceptor inhibition. Thus, eCBs could elicit a strong DA response in the nucleus accumbens and striatum. The effect could possibly overpower the motivational value of sex steroids and increase the likelihood of mate-seeking behavior [67].

On average, Δ^9^-THC affects males and females differently. This is not to say that there is not a large variation in response within the sexes, but there have been enough differences shown to suggest gonadal steroid modulation of exogenous cannabinoid reward. After showing that CB_1_ agonists induce stronger analgesic and motor suppressing effects in female rats than in male rats, Craft and colleagues investigated whether activational effects of gonadal hormones were responsible for these differences. In males, T attenuated the motor effects of Δ^9^-THC. In females, estrogen was linked to increased antinociception. OVX females showed less analgesia in response to Δ^9^-THC than OVX females given estrogen. In addition, intact estrous females showed more antinociception than diestrous females [102]. Likewise, Fattore and colleagues determined that female rats found the CB_1_ agonist WIN55,212-2 (WIN) more rewarding than male rats. Compared to male rats and OVX females, female rats showed faster acquisition of WIN self-administration and higher overall drug intake. However, gonad-intact female rats showed faster extinction for WIN self-administration. One explanation for Fattore's work is that there is a higher hedonistic value on cannabinoids for females [103]. On the other hand, estrogen may attenuate the disruptive effects of Δ^9^-THC on learning, leaving female rats less affected by a negative side effect [104]. It is possible that the greater response to Δ^9^-THC seen in female rats is due to estrogen modulation of DA signaling in the ventral tegmental area and nucleus accumbens. However, precise interactions between cannabinoids and estrogen are not well understood [67].

## 5. Insights in the Progression of the Spermatogenesis and the Acquisition of Sperm Functions

The suppression of LH levels in marijuana smokers as well as in animal models is related to the impairment of hypothalamic GnRH secretion ([51, 105, 106] for reviews). However, the presence of eCBs in reproductive fluids [1, 107, 108] and the ubiquity of testicular endocannabinoid activity are critical for the activity of Sertoli and Leydig cells, for germ cells progression and sperm quality [51, 105, 106].

Interstitial Leydig cells were the first target of CB_1_ activity to be identified [70, 109]. Such CB_1_ dependent modulation has been confirmed in *CB*_1_^−/−^ mice, where a decreased number of Leydig cells [110] and low estrogen levels [44] have been observed. Consistingly, also in nonmammalian vertebrates *CB*_*1*_ mRNA [111], but not CB_1_ protein [59], has been localized in interstitial compartment. In the germinal compartment AEA reduces the spermatogenetic output by inducing apoptosis of Sertoli cells [41], a process reversed by FSH-dependent activation of aromatase and by E_2_-dependent activation of FAAH [41, 42]. The involvement of endocannabinoid signaling in the progression of spermatogenetic stages has been only recently elucidated. In *Rana esculenta* increasing levels of CB_1_ and FAAH have been detected in postmeiotic stages [59, 60], whereas *NAPE- PLD* has been detected by *in-situ* hybridization in Leydig cells and mitotic and early-meiotic stages [111]. In mice, CB_1_, CB_2_, and TRPV1 fluctuate in a stage specific manner [32, 109]. During the first spermatogenetic wave transcriptional downregulation of *CB*_*1*_ has been observed as soon as meiotic events occur [109] whereas the expression peak has been observed in postmeiotic stages [32, 109]. Besides the control of sperm function required for the fertilization (i.e., sperm motility, capacitation, and acrosome reaction) CB_1_ activity in chromatin remodeling during the spermiogenesis has been recently reported [43–45]. Interestingly, CB_2_, the receptor with higher affinity to 2-AG than CB_1_, is highly expressed in mitotic/meiotic stages and the protein is retained in residual body at the end of the spermiogenesis [32], indicating CB_2_ participation in meiotic progression. Consistently to the above observations, the levels of eCBs, especially 2-AG, decrease throughout the progression of spermatogenesis, being higher in the spermatogonia and reaching minimal level in spermatids [32]. Lastly, an intriguing matter of debate is the high expression of *TRPV1* observed in meiotic stages [32] and the massive germ cell depletion observed in mice lacking the receptor [112]. Thus, a possible role in the protection of meiotic stages has been postulated for TRPV1.

In such a context, the gonadal activity of neurohormones such as GnRH might be critical. In human testes two GnRH molecular forms and two GnRH-Rs have been detected [47, 113, 114], with *GnRH-RII* gene postmeiotically expressed in round and elongating spermatids. Beside a central query to be resolved is whether these transcripts are functional in sperm the mRNA levels of *GnRH-I, GnRH-II, GnRH-R, cytochrome P450 side-chain cleavage* (*CYP11A1*), and *3beta-hydroxy-steroid dehydrogenase type 2 *enzyme (*HSD3B2*) as well as the intratesticular T levels are significantly increased in patients with spermatogenic failure [115] indicating that testicular GnRH may locally act to regulate spermatogenesis and steroidogenesis in humans. Once again, data obtained in nonmammalian vertebrates as well as in mollusks confirmed the involvement of local GnRH in processes such as Sertoli-Leydig cells communication, estradiol dependent spermatogonia proliferation, and sperm release [47, 48, 74, 116, 117] whereas evidences in frogs and rats suggest the participation in sperm functions related to fertilization [111, 118]. Only recently has AEA-dependent modulation of local GnRH system been provided in amphibian testes. In fact, during the annual sexual cycle eCBs, via CB_1_ activation, modulate GnRH activity in frog testes in a stage dependent manner [111]. When the upsurge of a new spermatogenetic wave occurs (February), *in vitro *AEA treatment specifically upregulates *GnRH-II *and *GnRH-RIII *mRNA and downregulates *GnRH-RII*. Conversely, in postreproductive period (June), *in vitro *AEA treatment significantly decreases *GnRH-I *and *GnRH-RII *mRNA, whereas it stimulates the transcription of *GnRH-II *and* GnRH-RI*. *GnRH/GnRH-R* localization in frog testes clearly indicates a functional distribution with a GnRH-I/GnRH-RII system mainly involved in the control of germ cell progression and Leydig/Sertoli cell communication and a GnRH-II/GnRH-RII system mainly involved in the control of sperm functions [111]. Thus, the differential AEA-dependent modulation of hypothalamic and testicular GnRH systems may reflect the functional divergence of GnRH molecular forms in testes. In such a picture, TRPV1 signaling should be also considered since, in postreproductive period, the activation of TRPV1 modulates the transcription of testicular *GnRHs *and of *GnRH-RI* and *GnRH-RII*, but in an opposite way compared to that of AEA ([119] in this issue).

Focusing on sperm functions, recently, a fertilization strategy adapted mechanism (external or internal fertilization) has been characterized for the control of sperm motility. In amphibians, exhibiting fertilization in aquatic environment, endocannabinoid activity in cloacal fluid may keep SPZ in a quiescent stage; the addition of CB_1_ antagonist SR141716A [120] and/or the dilution of cloacal fluid soon increase SPZ motility, in a fashion that mimics the quick activation of SPZ in the aquatic environment during the mating [59]. Such a “dilution-activating mechanism,” in mammals adapted into a 2-AG functional gradient inside the epididymus, the anatomical structure in which SPZ acquire the motility. High 2-AG level has been measured in the caput where SPZ are immotile whereas low level has been detected in the cauda, where SPZ acquire the ability to become motile [34, 121]. Accordingly, (1) the SPZ of *CB*_1_^−/−^ mice early acquire the motility in the caput epididymus [62], (2) the pharmacological inactivation of CB_1_ drives the same effects observed in knockout animals, and (3) the administration of EMT inhibitors results in the falling down of cauda motile SPZ in normal mice [34].

A tight control of eCBs levels in SPZ and seminal plasma is required to assure the correct progression of multiple steps involved in the fertilization process. In fact, it has been reported that in *FAAH *null mice (*FAAH* ^−/−^) elevated AEA levels [122] impair the sperm fertilizing ability and motility, and the administration of HU-210, a synthetic analogue of Δ^9^-THC, to rats has adverse effects on both spermatogenesis and sperm motility, suggesting that heightened AEA signaling in the male reproductive tract compromises some sperm cells features [29]. Recently, low 2-AG or AEA levels were measured in seminal plasma of infertile men [35, 123], thus suggesting a key role of eCBs in the acquisition of sperm functions and opening new perspectives in the treatment of male infertility.

The importance to keep AEA content at physiological concentrations in cells, tissues, and fluids involved in male and female reproductive events might be related to the existence of an eCBs gradient. In this context, several papers highlighted the involvement of eCBs signaling in the spatiotemporal control of sperm-egg fusion [26, 63, 108, 124]. Analogously to human menstrual cycle phases [125, 126], fluctuations of AEA levels, in combination with sex hormones oscillations, were detected in the various stages of bovine oestrus cycle [108], strengthening the idea that oviductal AEA content is crucial to avoid impairments in the normal sperm-oocyte interaction.

## 6. eCBs and Pregnancy: A Focus on Placentation and Parturition

In the past few decades, a large amount of evidence has demonstrated that endocannabinoid signaling via cannabinoid receptors is an important player in various female reproductive events, including sperm-egg fusion as fertilization, preimplantation development of embryos and their timely transport from the oviduct into the uterus, attainment of uterine receptivity, embryo-uterine crosstalk during implantation and decidualization, trophoblast differentiation and placental development, and initiation of parturition. In this section, we will briefly introduce the involvement of endocannabinoid signaling in early pregnancy events, with a focus on its pathophysiological significance during trophoblast development and placental formation as well as the labor onset.

### 6.1. Endocannabinoid Signaling in Early Pregnancy Events

In mammals, the beginning of a new life is seeded at fertilization. The fertilized egg undergoes serial cell divisions to form the 2-cell embryo, 4-cell embryo, 8-cell embryo, morula, and eventually the blastocyst with the first two differential cell lineages, the inner cell mass (ICM), and the trophectoderm [127–129]. During the past two decades, molecular and genetic studies have demonstrated that the ECS is tightly associated with early pregnancy events [130]. For example, cannabinoid receptors are expressed in the preimplantation mouse embryo, as well as in the oviduct and uterus. In mice, *CB*_*1*_ mRNA is primarily detected from the four-cell embryo through the blastocyst stages, while *CB*_*2*_ mRNA is present from the zygote through the blastocyst stages [8, 9, 131]. These results indicate that preimplantation embryo is a potential target for endocannabinoid signaling. Activation of CB_1_ by cannabinoid ligands interferes with preimplantation embryo development in culture [9]. On the other hand, asynchronous preimplantation embryo development is also observed in mice lacking CB_1_ [131]. This pharmacological and genetic evidence pointed toward a tightly regulated endocannabinoid signaling during preimplantation embryo development [9, 11, 131].

During early pregnancy, another critical event occurring in parallel with preimplantation embryo development is the timely transport of preimplantation embryos from the oviduct into the uterus. In mice, embryos at the late morula or early blastocyst stage enter the uterus, where they develop and differentiate to gain implantation competency, escape from the zona pellucida, and implant into the receptive uterus. Therefore, normal oviductal embryo transport is one of the prerequisites for on-time implantation. In *CB*_1_^−/−^, a large portion of embryos are retained in the oviduct on day 4 of pregnancy and thus fail to initiate on-time implantation [132]. Moreover, wild-type mice treated with methanandamide, a CB_1_ agonist, also exhibit a similar phenomenon, collectively suggesting that a tonic endocannabinoid signaling is essential for normal embryo transport from the oviduct into the uterus prior to blastocyst implantation. The endogenous levels of AEA, one of the primary endocannabinoid, are maintained by its synthesis and degradation activity. In this respect, *FAAH* ^−/−^ mice exhibit an elevated level of AEA in the oviduct during early pregnancy, accompanied with a derailed oviductal embryo transport [133]. Thus, an aberrant cannabinoid signaling impairs the oviductal transport of embryos, preventing on-time implantation [132, 133]. This finding is clinically relevant to human ectopic pregnancy, since high AEA levels and aberrant expression of FAAH and CB_1_ in fallopian tubes have been observed in women with ectopic pregnancy [130, 134, 135]. Synchronized embryo development to blastocyst and uterine differentiation to receptive state are important for successful implantation. In the mouse, at pregnant day 1 to day 4 (day 1 = vaginal plug), the ovarian hormones estrogen and progesterone control the uterine undergoing from prereceptive to receptive stage. In this respect, lower levels of AEA in the receptive uterus and at the implantation site have been observed in contrast to its high levels in the nonreceptive uterus [13, 131]. Moreover, the CB_1_ expression in activated blastocyst is significantly lower than that in dormant blastocysts [12, 131]. These observations suggest a biphasic role of endocannabinoid signaling in synchronizing trophoblast differentiation and uterine preparation to the receptive state for implantation. Also in female rats, ovarian hormones operate in conjunction with the blastocyst intrinsic programme, in order to regulate the synthesis of AEA in a specific manner during the crucial reproductive events that may compromise pregnancy outcome [136]. However, the interaction between lysophosphatidic acid, prostaglandins, and ECS during the window of implantation in the rat uterus has also been reported [137]. Indeed, employing delayed implantation model, previous studies have further demonstrated that AEA at low level renders the blastocyst competent for implantation viaactivating mitogen-activated protein kinase (MAPK) signaling, whereas at a higher concentration it inhibits calcium channel activity and blastocyst reactivation for implantation [12]. This finding has high clinical relevance, since the circulating level of AEA is well associated with pregnancy outcome in women with threatened miscarriage [36, 138]. Taken together, endocannabinoid signaling is an important player directing the normal preimplantation embryo development, activation, and uterine differentiation during the peri-implantation embryo-uterine dialogue.

### 6.2. Endocannabinoid Signaling Regulates Trophoblast Development and Placentation

With the initiation and progression of implantation and decidualization, trophectodermal epithelium, the wall of spherical blastocyst, will further develop into the extraembryonic tissues and eventually form the placenta. In mice, while the mural trophectoderm penetrates the uterine stromal, forming primary trophoblast giant cells, the polar trophectoderm, adjacent to the ICM, continues to proliferate and forms the ectoplacental cone (EPC) of the early conceptus and the extraembryonic ectoderm [129, 139]. Thereafter, the extraembryonic ectoderm develops to form the chorionic epithelium, which will be further fused with the allantois. Soon after, the chorionic trophoblast and its associated fetal blood vessels undergo extensive villous branching to create a functional mature placenta [140, 141]. Placenta serves as an interface for the exchange of nutrients, gases, and wastes between the maternal and fetal compartments. Moreover, placenta can secrete many hormones and growth factors conducive to the success of pregnancy establishment and maintenance [140, 141].

Increasing evidence suggests that the placenta is also a target of endocannabinoid signaling. In mice, CB_1_ and FAAH are expressed in the EPC, and later in the spongiotrophoblast cells [142]. *CB*_1_^−/−^ placentas exhibit compromised spongiotrophoblast development with reduced expression of Mash2 and trophoblast-specific protein *α* (Tpbpa). This reduced population of Tpbpa positive trophoblast cells is due to an attenuated proliferation of spongiotrophoblast cells in the absence of CB_1_ receptors [142]. This is consistent with the observations that CB_1_/CB_2_ null mutant trophoblast stem (TS) cells show remarkably slower cell proliferation compared with that in wild-type TS cells [142, 143]. It has been further demonstrated that endocannabinoid signaling regulates trophoblast cell proliferation via PI3 K/AKT signaling pathway [142]. Endocannabinoid signaling is also operative during human placental development, since CB_1_, FAAH, and NAPE-PLD have been demonstrated to be expressed in human placentas [144–147]. For example, CB_1_ receptors are present in all layers of the membrane, with particularly strong expression in the amniotic epithelium and reticular cells. Moderate expression is observed in the chorionic cytotrophoblasts. Moreover, FAAH is highly expressed in the amniotic epithelial cells, chorionic cytotrophoblast, and maternal decidua layer [145]. Besides, emerging evidence suggests that the levels of CB_1_, FAAH, and NAPE-PLD in first trimester placentas are highly associated with the term pregnancy outcomes. The expression levels of CB_1_ and FAAH are significantly lower or even absent, whereas the *NAPE-PLD* mRNA expression is aberrantly higher in spontaneous miscarriage women [20]. Higher level of AEA is also detected in plasma of nonviable pregnancies than in viable pregnancies [147]. Most recent study further demonstrates that aberrant endocannabinoid signaling plays an important role in the pathophysiology of preeclampsia. The placental expression of NAPE-PLD is significantly higher in preeclamptic pregnancies, while FAAH exhibits an opposite result [148]. Moreover, AEA and Δ^9^-THC have been shown to be able to inhibit human trophoblast BeWo cell proliferation and the transcription of genes involved in growth and apoptosis [138, 149]. These findings reinforce the notion that a tightly regulated endocannabinoid signaling is conducive to normal trophoblast development and placentation in humans.

### 6.3. Endocannabinoid Signaling Is Operative during Labor Onset

Preterm birth is defined as the birth of a baby which is less than 37 weeks of gestational age in humans [150]. In the world, 15 million babies are born prematurely [151]. Preterm birth is among the top causes of death in infants worldwide, which is the greatest health burden associated with pregnancy and childbirth [152]. Preterm labor may be caused by many factors, for example, genetics, infection, chemical substances, environmental contaminant or other factors [153–157], but the cause of preterm birth in many situations is elusive and unknown.

Progesterone and corticotropin-releasing hormone (CRH) are the most important mediators of labor. Progesterone has an essential and multifaceted role in the maintenance of myometrial quiescence during pregnancy and its withdrawal induces labor. The functions of progesterone are mediated by the nuclear progesterone receptors (PR-A and PR-B) in myometrial cells [158]. Progesterone has been advocated for the prevention of preterm labor [159]. Treatment with progesterone reduces the rate of spontaneous early preterm delivery in the midgestation period in women [159, 160]. CRH also has a critical role in pregnancy and labor, which is produced by the placenta during pregnancy [161–163]. CRH acts on the fetal pituitary-adrenal axis and directly on myometrial cells to facilitate labor, which determines the length of gestation and the timing of parturition and delivery. In this respect, previous studies have demonstrated that endocannabinoid signaling can modulate the activities of the hypothalamic-pituitary axis [77, 164–166] and thus is associated with normal onset and duration of labor in both mice and women [19, 167].

In mice, as described above, loss of CB_1_ impairs the normal oviductal embryo transport, leading to deferral of on-time embryo implantation [132]. Therefore, it was generally thought that the labor onset would accordingly be delayed. However, surprisingly, the day of birth of CB_1_ null mutant females is almost one day earlier than that in wild-type mice [167]. Similar premature birth can be induced in wild-type mice receiving CB_1_-selective antagonist SR141716, but not a CB_2_-selective antagonist SR144528 [120, 168]. The levels of progesterone and estrogen are largely alerted in the CB_1_deficient mice. An early drop of serum progesterone levels is observed on day 19 in the CB_1_ null mutant mice, while the estrogen level increases on days 16–18. Subsequent analysis further reveals that cytochrome P450 aromatase and 17*β*-HSD7, which primarily contribute to ovarian estrogen biosynthesis during gestation in mice, are upregulated in CB_1_ null ovaries, whereas levels of 20*α*-HSD, which metabolize progesterone into biologically inactive 20*α*-dihydroprogesterone, are substantially increased in CB_1_ null mutant ovaries on day 19 of pregnancy. The premature birth in mice lacking *CB*_*1*_ can be restored by subcutaneous injection of progesterone on day 18. This finding suggests that endocannabinoid signaling is essential for the maintenance of normal progesterone/estrogen ratio prior to the onset of parturition. Another interesting finding is that loss of CB_1_ overrides cyclooxygenase- (COX-) 1 deficiency-induced delayed parturition and remarkably improves the survival rate of newborn pups. These results suggest that CB_1_ signaling has a unique role in regulating normal parturition that is independent of COX-1-derived prostaglandin F2*α*, but CB_1_ deficiency can correct the effects produced by COX-1 deficiency [167]. There is evidence that eCBs via CB_1_ can upregulate *COX-2* expression and thus prostaglandin E_2_ production in human gestational membranes during late pregnancy [169]. Prostaglandin E and F have an important function to regulate uterine contractions in labor, and the function of prostaglandin was through prostaglandin receptor expressed in myometrial tissue [170]. It remains to be determined whether COX-1 deficiency-induced delayed parturition is associated with aberrant cannabinoid-CB_1_ signaling in mice. In addition, loss of CB_1_ induces aberrant CRH-driven endocrine activities leading to preterm labor in mice, Antalarmin hydrochloride, a selective CRH antagonist, is able to restore the normal parturition timing in CB_1_ deficient mice, and enhanced corticosterone activity on days 14–18 induces preterm birth with impaired fetal growth in wild-type mice. These observations show the concept that CB_1_ signaling is crucial for maintaining normal CRH- corticosterone activities and onset of labor in mice [167].

In women, the chronic use of marijuana is often associated with fetal abnormalities and early pregnancy termination [36, 37, 133]. Plasma AEA levels have been shown to be associated with onset of labor. Plasma AEA levels are significantly increased in laboring term than those in nonlaboring term [19, 171, 172]. Meanwhile, a significantly higher expression of CB_1_ has been observed in placental villous from nonlaboring compared to laboring women 173]. This finding indicates that the higher AEA level and lower placental CB_1_ expression are essential for the timely onset of labor.

Collectively, endocannabinoid signaling is crucial for the normal initiation of parturition. Epidemiological studies should pay a close attention to *CB*_*1*_ or *FAAH* gene polymorphism or mutation in women with preterm labor in clinical practice.

## 7. Closing Remarks 

In the past few years ECS has emerged as an essential player in male and female reproduction. Nowadays, eCBs together with their synthesizing and degrading enzymes, EMT, and molecular targets have been identified in reproductive cells, organs, and fluids of invertebrates, vertebrates, and mammals, highlighting the key role played by these endogenous compounds in reproduction processes along the evolutionary axis. Therefore, it comes out that the disruption of the normal physiological action of the ECS impairs the function of the male and female reproductive system and that altered AEA and/or 2-AG content is crucial during the various stages of procreation with relevant and interesting implications in the therapeutic exploitation.
